# Mediation of gaseous emissions and improving plant productivity by DCD and DMPP nitrification inhibitors: Meta-analysis of last three decades

**DOI:** 10.1007/s11356-023-26318-5

**Published:** 2023-03-16

**Authors:** Muhammad Aammar Tufail, Muhammad Irfan, Wajid Umar, Abdul Wakeel, Ruth A. Schmitz

**Affiliations:** 1 Institute for Microbiology, Christian-Albrechts-University Kiel, Kiel, Germany; 2 Soil and Environmental Sciences Division, Nuclear Institute of Agriculture (NIA), Tandojam, Pakistan; 3 Institute of Environmental Science, Hungarian University of Agriculture and Life Sciences, Gödöllő, 2100 Hungary; 4 Institute of Soil and Environmental Sciences, University of Agriculture Faisalabad, Faisalabad, Pakistan

**Keywords:** GHG emission, Nitrification inhibitors, DCD, DMPP, Nitrogenous gasses, Precision agriculture

## Abstract

Nitrification inhibitors (NIs), especially dicyandiamide (DCD) and 3,4-dimethylpyrazole phosphate (DMPP), have been extensively investigated to mitigate nitrogen (N) losses from the soil and thus improve crop productivity by enhancing N use efficiency. However, to provide crop and soil-specific guidelines about using these NIs, a quantitative assessment of their efficacy in mitigating gaseous emissions, worth for nitrate leaching, and improving crop productivity under different crops and soils is yet required. Therefore, based upon 146 peer-reviewed research studies, we conducted a meta-analysis to quantify the effect of DCD and DMPP on gaseous emissions, nitrate leaching, soil inorganic N, and crop productivity under different variates. The efficacy of the NIs in reducing the emissions of CO_2_, CH_4_, NO, and N_2_O highly depends on the crop, soil, and experiment types. The comparative efficacy of DCD in reducing N_2_O emission was higher than the DMPP under maize, grasses, and fallow soils in both organic and chemical fertilizer amended soils. The use of DCD was linked to increased NH_3_ emission in vegetables, rice, and grasses. Depending upon the crop, soil, and fertilizer type, both the NIs decreased nitrate leaching from soils; however, DMPP was more effective. Nevertheless, the effect of DCD on crop productivity indicators, including N uptake, N use efficiency, and biomass/yield was higher than DMPP due to certain factors. Moreover, among soils, crops, and fertilizer types, the response by plant productivity indicators to the application of NIs ranged between 35 and 43%. Overall, the finding of this meta-analysis strongly suggests the use of DCD and DMPP while considering the crop, fertilizer, and soil types.

**Graphical Abstract:**

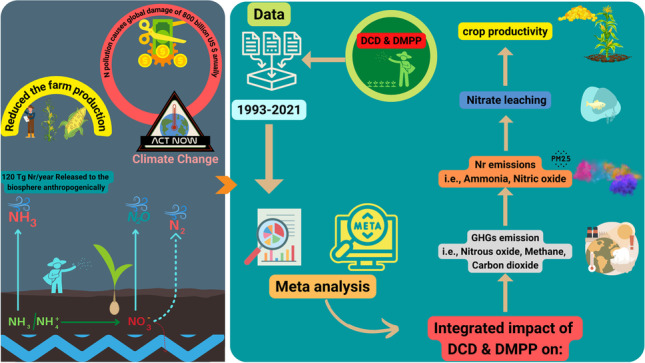

**Supplementary Information:**

The online version contains supplementary material available at 10.1007/s11356-023-26318-5.

## Introduction

Modern agriculture largely depends on synthetic nitrogen (N) fertilizer for sustaining crop productivity and ensuring global food security. However, mitigating climate change and improving food security are two of the world’s most challenging issues (Shakoor et al. 2021). Since the invention of the Haber–Bosch process, N use in agriculture has increased substantially to feed the rapidly expanding population (Billen et al. 2013; Erisman et al. 2008; Smith et al. 2020). By 2050, the world’s population is expected to touch 10 billion (Shakoor et al. 2020), and global N consumption has been projected to escalate from 142 to 169% by 2050 to achieve a 100–110% increase in crop yields (IFA, 2013). Excessive use of N fertilizers causes severe N losses into the environment, thereby reducing applied N’s efficiency to 20–50% (Ahmed et al. 2017). Overuse of N fertilizers accompanied by low N use efficiency (NUE) results in substantial monetary and environmental costs. For instance, Sutton et al. (2013) estimated about 800 billion $US as global damage because of N pollution annually. The amount of reactive N (Nr) released into the biosphere through anthropogenic means is prodigious and is estimated to be 120 Tg per year, which is twice the N fixed by all-natural terrestrial processes, i.e., 63 Tg per year (Fowler et al. 2013; Sánchez-Vicente et al. 2019). Higher Nr in atmosphere, aquatic and terrestrial systems are creating serious environmental consequences such as global warming, greenhouse gas-driven climate change, nitrate contamination, eutrophication of freshwater resources, soil acidification, and loss of biodiversity (Liu et al. 2013; Tilman and Isbell 2015; Zhu et al. 2016) and negative impacts on human health by deteriorating air quality (Xu et al. 2017).

Globally, N recovery by major cereal crops, i.e., rice, wheat, and maize, is notoriously low and often remains below 50% during the first season of N application (Coskun et al. 2017; Herrera et al. 2016; Shahzad et al. 2019), while only < 10% of residual N is recovered during subsequent years (Congreves et al. 2021). Gaseous emissions, including ammonia (NH_3_), nitric oxide (NO), nitrous oxide (N_2_O), and dinitrogen (N_2_), are the primary routes of N losses (Fowler et al. 2013; Xia et al. 2017), causing colossal loss of resource investment in addition to environmental pollution. Nitrous oxide has become the leading stratospheric ozone-depleting gas during the twenty-first century, and its concentration in the atmosphere is continuously increasing at the rate of 0.2–0.3% per annum (IPCC, 2014). About 60–80% of global anthropogenic N_2_O emissions and 10–12% of total greenhouse gas emissions are attributed to agriculture (Turner et al. 2015; Zhang et al. 2020). Hence, eco-friendly and cost-effective strategies to reduce Nr and improve N resource efficiency are urgently required to address environmental problems without yield penalties.

The Nr losses from agroecosystems originate from the deprotonation of ammonium (NH_4_^+^) to ammonia (NH_3_). This process is governed by soil pH, soil organic matter, soil texture, temperature, moisture, N application rate, and several microbial-mediated nitrification and denitrification reactions (Chen et al. 2015). Nitrification, oxidation of NH_3_ to nitrate (NO_3_^−^), is catalyzed by the ammonia-oxidizing bacteria (AOB), ammonia-oxidizing archaea (AOA), and nitrite-oxidizing bacteria (NOB). The nitrification process is initiated by AOB (*Nitrosomonas* and *Nitrosococcus* spp.) which oxidizes NH_3_ to NH_2_OH (hydroxylamine) via ammonia monooxygenase enzyme, and then NH_2_OH is oxidized to NO_2_^−^ by the enzyme hydroxylamine oxidoreductase. Finally, the NO_3_^−^ is produced by NOB (*Nitrobacter* spp.) through the enzyme nitrite oxidoreductase (Daims et al. 2016). The denitrification process is also catalyzed by a diverse set of bacteria, archaea, and fungi (*Nitrosospira*, *Chaetomium*, and *Fusarium*) which reduced NO_3_^−^ to NO_2_^−^, NO, N_2_O, and N_2_ (Hayatsu et al. 2008; Rex et al. 2019).

Nitrification inhibitors (NIs) have been recognized as promising tools to mitigate Nr pollution associated with increased N inputs in cropping systems worldwide (Sha et al. 2020). NIs can provide substantial agronomic, economic, and environmental benefits. They potentially minimize the gaseous N losses and improve NUE through deactivating ammonia monooxygenase (AMO), thus limiting the conversion rate of NH_3_ to NO_3_^−^ (Fu et al. 2020). NIs are products that inhibit the bacterial oxidation of NH_4_^+^ to NO_2_^−^ in soil, so maintaining N as NH_4_^+^-N (Benckiser et al. 2013) can reduce leaching, denitrification, and emission of N_2_O (Hu et al. 2015), as NIs retain soil N in a less mobile form NH_4_^+^, hence providing a better opportunity to plants for more N uptake (Kim et al. 2012; Wang et al. 2021).

Dicyandiamide (DCD) and 3,4-dimethylpyrazole phosphate (DMPP) are the most widely used NIs in agriculture globally. Both compounds have different chemical characteristics and mechanisms for restricting nitrification (Barth et al. 2001). Diverse climate zones, ecosystems, soil types, and planting systems are the critical influential factors resulting in large disparities in the performance of these compounds. Likewise, inhibitor type, soil pH, organic matter content, and N rate are the key factors for retarding the nitrification process (Sha et al. 2020). The advantages and effectiveness of NIs in improving crop yields and NUE, minimizing N losses via NO_3_^−^ leaching, greenhouse gas emissions, and NH_3_ volatilization (Afshar et al. 2018; Sun et al. 2015), have been widely reported. Akiyama et al. (2010) reported that N_2_O emissions from N fertilizer could be reduced by 38% by using NIs, while Gilsanz et al. (2016) found that DCD and DMPP are effective in reducing N_2_O emissions by 42 and 40%, respectively. Furthermore, NIs enhance the activity of methane monooxygenase and influence soil carbonate hydrolyzation thereby reducing carbon dioxide (CO_2_) and methane (CH_4_) emissions due to soil acidification (Fan et al. 2019).

Integrated assessments (using a meta-analysis approach) could provide an opportunity to summarize the findings from available studies to formulate a tangible estimate regarding the impact of NIs on the environment and crop yields. Several meta-analyses on the use and efficacy of NIs in agriculture on an individual basis, for example, crop yield and productivity (Hu et al. 2014; Yang et al. 2016b), NUE (Abalos et al. 2014), emission factor (Gilsanz et al. 2016), and NH_3_ volatilization (Pan et al. 2016), have previously been conducted. Nevertheless, still little is known on how NIs simultaneously affect greenhouse gas emissions, Nr emissions, NO_3_^−^ leaching, plant productivity, and soil inorganic N contents in agricultural soils. Therefore, we performed the first comprehensive global meta-analysis study to fulfill this knowledge gap. This study was aimed primarily at investigating the efficacy of NIs particularly DCD and DMPP on greenhouse gas emissions (CO_2_, CH_4_, and N_2_O), Nr emissions (NH_3_ and NO), NO_3_^−^ leaching, plant productivity (NUE, plant biomass, grain-N content, and plant-N update), and soil inorganic N content, simultaneously.

## Materials and methods

### Database search and selection criteria

Metadata was obtained following the PRISMA reporting guidelines (Liberati et al. 2009). A literature search was conducted in March 2021 using the SCOPUS® database (http://www.scopus.com) and the Web of Science® database (https://webofknowledge.com/). Articles published in scientific journals in only the English language were retrieved using the following keyword combination: “nitrification inhibitor” AND (“DCD” OR “DMPP”). The Boolean truncation “*” character is included in combination to ensure variations of the words, such as inhibitor or inhibitors. The logical operator “AND” was used to refine the articles that contain words written on both sides of the operator. Articles found through the cross-reference citations from review and research papers were also retrieved.

### Study selection

Metadata searches from both databases yielded 1366 articles, 714 of which were left after duplicate removal. The following eligibility criteria for the study selection were predefined to eliminate publication bias:

1. The study should have demonstrated the effects of at least one nitrification inhibitor (DCD or DMPP).
2. Studies investigating any parameters from gaseous emission, N leaching, plant productivity, and/or soil inorganic-N were selected.
3. Studies investigating the combined effect of NIs were excluded.

**Fig. 1:**
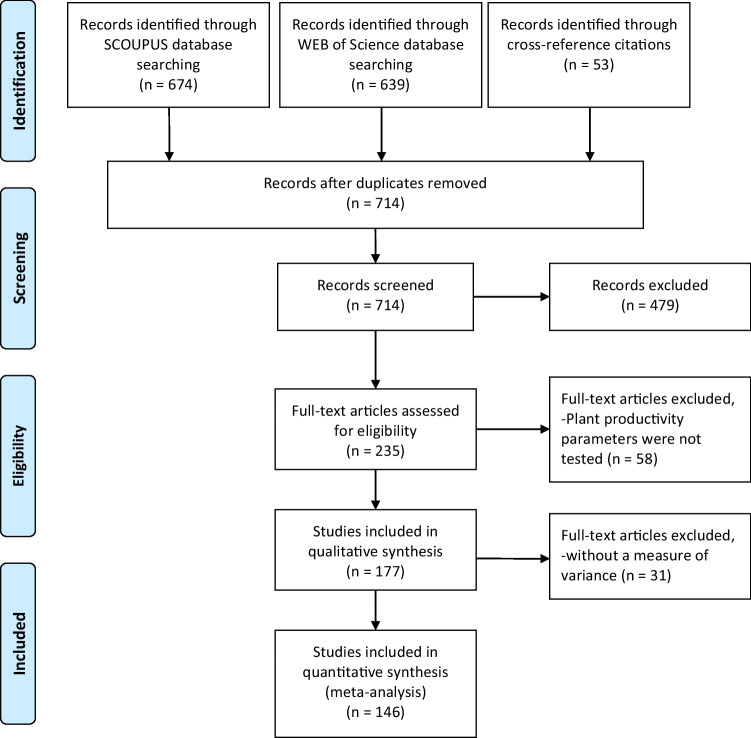
Preferred reporting items for systematic reviews and meta-analyses (PRISMA) flow diagram for the meta-analysis

The studies not fulfilling the above criteria were excluded from this analysis. If any of the traits were measured over time, the data only for the final measurements were included. Out of the 235 articles assessed for eligibility, 146 articles fulfilling our criteria were selected (Fig. 1, Table S1). The selected papers spanned almost three decades, from 1993 to 2021.

### Data extraction

Data including treatment means, sample size (number of replications, *n*), and standard deviation were extracted from each study. The standard errors (SE) reported in some studies were converted into standard deviations (SD) using the following equation: SE = SD (*n*^−1/2^). The data from the graphs were digitized using Web Plot Digitizer (Ankit, 2020). Since multiple experiments from one study do not increase the dependence of meta-analysis on that study (Gurevitch and Hedges 1999), therefore, different treatments such as fertilizers or nitrification inhibitors in a given study were regarded as independent experiments and described in the study as separate data units. This technique increases the power of meta-analysis (Lajeunesse and Forbes 2003) and has been used in several meta-analyses (Dastogeer 2018; Mayerhofer et al. 2013; Mcgrath and Lobell 2013). Parameters related to gaseous emission (CO_2_, CH_4_, N_2_O, NH_3_, and NO), NO_3_^−^ leaching, crop productivity (grain N content, N uptake, N use efficiency (NUE), and biomass/yield), and soil inorganic N (NH_4_^+^ and NO_3_^−^) were collected from each study for different crop types, experiment types, fertilizer types, and soil texture and pH types.

### Meta-analysis

To estimate the effect size of DCD or DMPP treatment on gaseous emissions, NO_3_^−^ leaching, plant productivity, and soil inorganic-N as compared to control (without DCD and DMPP), log response ratio (ln*RR*) was calculated using the following formula: ln*RR* = ln (*V*_*ni*_/*V*_*c*_), where *V*_*ni*_ is the mean of nitrification inhibitor treatment and *V*_*c*_ is the mean of control treatment without nitrification inhibitor (Hedges et al. 1999). The ln*RR* was used as an effect size metric because log transformation of the parameter(s) reported in different units among studies maintains symmetry within the analysis (Borenstein et al. 2011). Furthermore, percent change (%*Δ*) in effect size was calculated from ln*RR*, i.e., %*Δ* = (exp.(ln*RR*) − 1*100). We calculated pooled variances using the “escalc” function in the metafor (version 2.4–0) package of the R environment (Viechtbauer 2010).

Before constructing the meta-analysis model, the heterogeneity (*Q*) test was performed to determine the choice of fixed or random/mixed effects model. Heterogeneity on the full dataset, including 366 observations, was highly significant (Cochran’s *Q* = 124,337.57, *df* = 649, *p* < 0.001), indicating that a random/mixed effects approach was guaranteed (Cochran 1954).

It is assumed that studies with low effect sizes are less likely to be published than studies with high effect sizes due to publication bias (Rothstein et al. 2006). On the other hand, Head et al. (2015) stated that *p*-curve analysis for publication bias is not the cause of no or less publication rather; they “play” around their data (e.g., selectively removing outliers, choosing different outcomes, and controlling for different variables) until it becomes significant. This bad practice is called *p*-hacking and is very common among researchers. Therefore, a *p*-curve analysis of selected studies was conducted to check the publication bias.

The synthesis produced by this meta-analysis is balanced based on the weight of each study, to maintain an equal contribution to the results produced by meta-analysis. This study used the inverse variance method to assign the weights using meta and metafor packages in R. The estimated pooled effect sizes produced by the meta-analysis with their 95% confidence intervals (95% CI) were presented in forest plots. The effect of DCD or DMPP was considered significant if 95% CIs did not coincide with the zero line (Augé et al. 2014). A positive value indicates an increase, whereas a negative value indicates a decrease in the nitrogenous gasses’ emission following the application of DCD or DMPP. Statistical analyses were performed in R environment (https://r-project.org/) using metafor (Viechtbauer 2010), meta (Schwarzer 2007), and ggplot (Wickham 2011) packages.

### Metadata

**Fig. 2:**
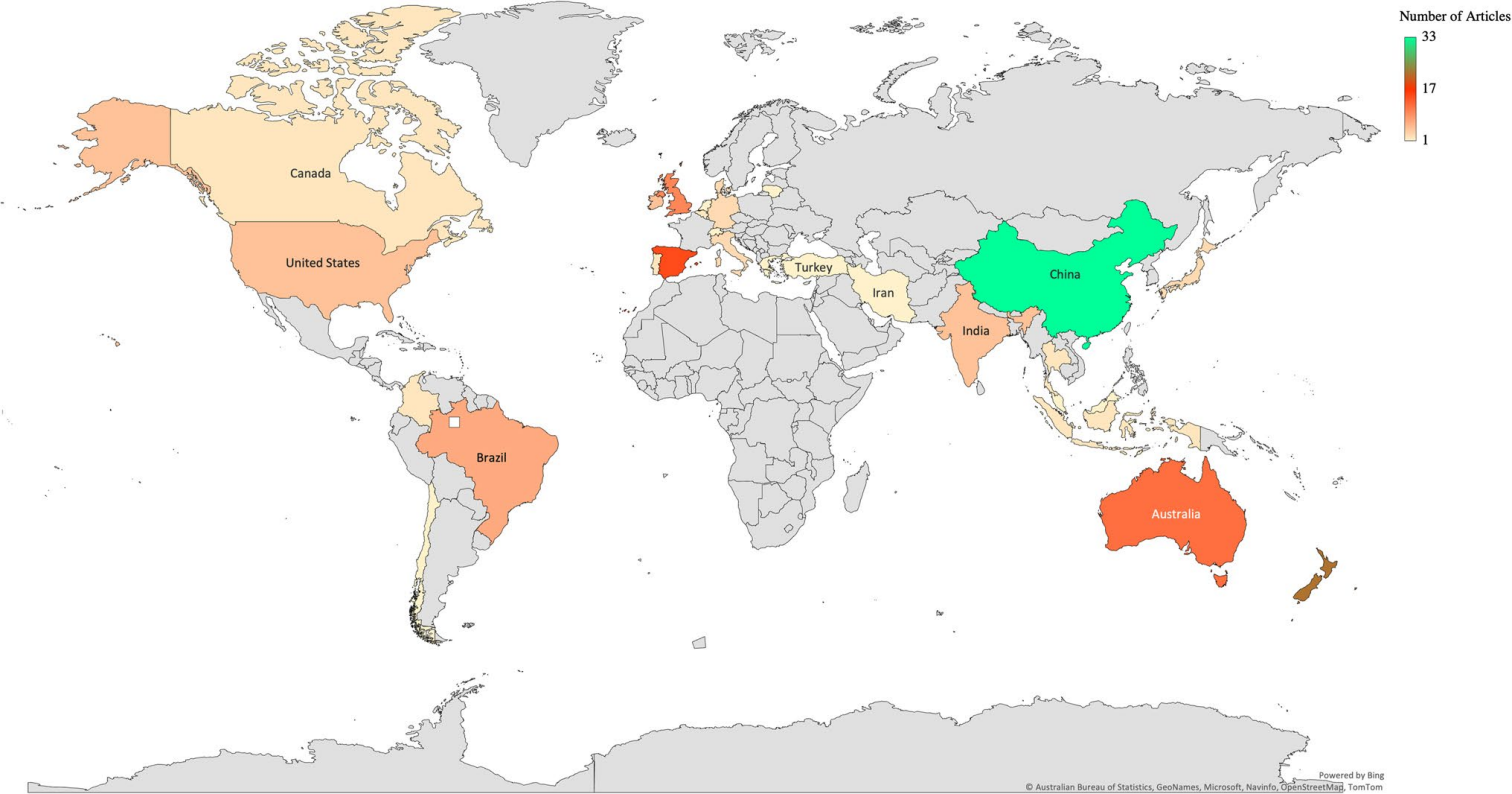
Location of the experiments obtained from the selected studies (146) used in this meta-analysis

Metadata was collected from 146 published scientific articles from 28 countries spanning between 1993 and 2021 (Fig. 2). A total of 650 observations (*k*) were obtained, including treatments without and using nitrification inhibitors.

### Publication bias

Out of 650 total observations, only 361 (55.54%) observations showed significant effect sizes at *p* < 0.05, and 328 (50.46%) observations showed significant effect sizes at *p* < 0.025. Studies showing nonsignificant results (*p* > 0.05) were excluded from the *p*-curve analysis. The *p*-curve plot shows that our data is significantly right-skewed and not flat, indicating an effect behind our data (Fig. S1). The estimated power of our studies in the meta-analysis is 99%, and the evident value is present, which shows the true effect size is present in the analysis.

## Results

### Overall effect of NIs

**Fig. 3:**
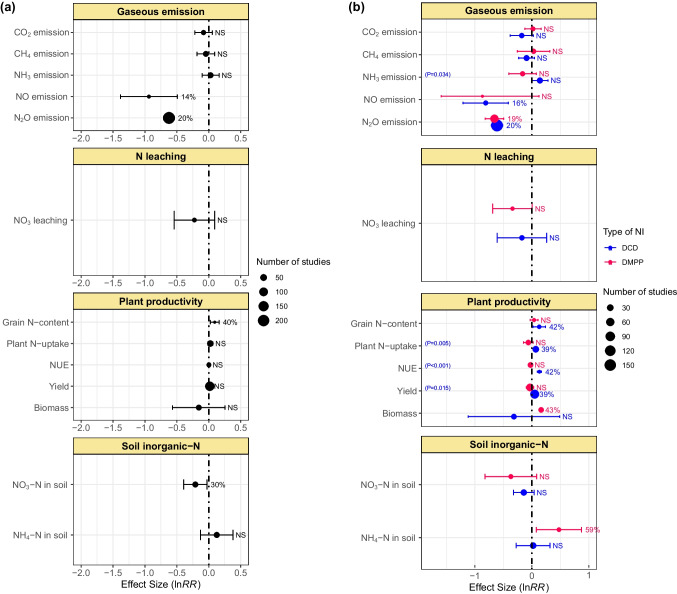
Overall effect of DCD and DMPP on gaseous emissions, N leaching, plant productivity, and soil inorganic-N. Error bars represent 95% CI. Variables are significant if error bars do not overlap with zero and are denoted in percent change (%) in effect size. Otherwise, NS shows a nonsignificant difference. Blue color represents the DCD, and red color represents DMPP treatments. The *p*-value inside each box denotes a significant difference between DCD and DMPP treatments, while nonsignificant differences show no *p*-values

The overall effects of NIs on the gaseous emissions, N leaching, plant productivity, and soil inorganic-N are presented in Fig. 3a. In general, the NIs remained ineffective in reducing CO_2_, CH_4_, and NH_3_ emissions from agricultural soils. However, they mitigated N_2_O and NO emissions by 20 and 14%, respectively. Plant productivity indicators (i.e., plant N-uptake, grain yield, and NUE) improved slightly, while grain N-content increased significantly (40%). On the other hand, no significant reduction in NO_3_^−^ leaching was observed with the use of the NIs. Nevertheless, the NIs effectively reduced the nitrification process, as indicated by the decline in NO_3_^−^-N content in soils by 30%, with a subsequent increase in soil NH_4_^+^-N.

The comparisons between the relative efficacy of the two NIs (DCD and DMPP) in minimizing N losses and improving crop productivity are presented in Fig. 3b. The DCD and DMPP, with reductions of 20 and 19%, respectively, remained equally effective in reducing N_2_O emissions from agricultural soils. However, DCD reduced NO emission by 16%, whereas the effect of DMPP was nonsignificant. The NIs showed a significantly (*p* = 0.034) different effect on NH_3_ emission: for instance, DCD slightly increased, while DMPP reduced NH_3_ emissions to some extent. Regarding plant productivity attributes, DCD increased grain N-content (42%), plant N-uptake (39%), grain yield (39%), and NUE (42%), whereas nonsignificant effects were observed when DMPP was applied. In the current study, both the NIs reduced soil NO_3_^−^-N content, but their effects remained nonsignificant. On the other hand, DMPP significantly increased soil NH_4_^+^-N (59%), while DCD did not exhibit any significant effect.

### Effect of NIs and crop type

**Fig. 4:**
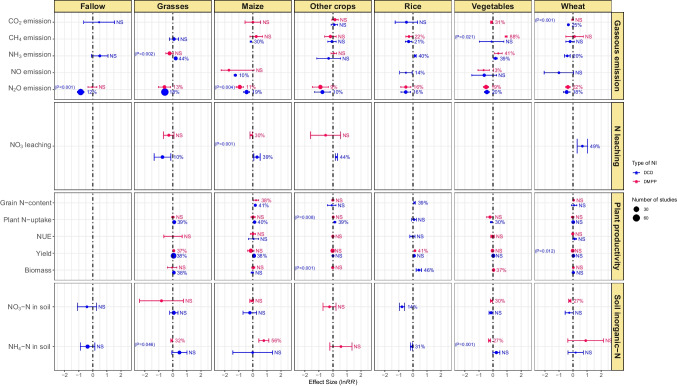
Effect of crop type on DCD and DMPP efficacy in gaseous emissions, NO_3_^−^ leaching, plant productivity, and soil inorganic-N. Variables are considered significant if error bars do not overlap with zero. Error bars represent 95% CI. Variables are significant if error bars do not overlap with zero and are denoted in percent change (%) in effect size. Otherwise, NS shows a nonsignificant difference. Blue color represents the DCD, and red color represents DMPP treatments. The *p*-value inside each box denotes a significant difference between DCD and DMPP treatments, while nonsignificant differences show no *p*-values

The effect of the NIs on CO_2_, CH_4_, NH_3,_ and NO emission was highly crop-type specific (Fig. 4). DCD significantly reduced the CO_2_ emission from the wheat field by 25%, CH_4_ emission from maize and rice fields by 30 and 21%, respectively, NH_3_ emission from wheat fields by 20%, and NO emission from rice and maize fields by 14 and 10%, respectively. On the other hand, DCD elevated NH_3_ emissions from vegetables and rice fields and grasslands by 39, 40, and 44%, respectively. DMPP reduced CO_2_ emission from vegetable fields by 31%, CH_4_ emission from rice fields by 22%, NH_3_ emission from wheat fields by 20%, and NO emission from vegetable fields by13%. DMPP application enhanced CH_4_ emission by 88% and NH_3_ emission by 41% from vegetable fields. Both the NIs proved highly effective in mitigating N_2_O emissions from all crop fields and crop types. DMPP curtailed N_2_O emission from wheat, rice, maize, vegetable fields, and grasslands by 21, 16, 11, 19, and 13%, respectively, while the corresponding decreases for DCD were 18, 16, 19, 20, and 18%, respectively (Fig. 4).

However, DCD and DMPP increased the biomass yield of rice by 39 and 41%, respectively, and the increase in yield in the former case was concomitant with a 46% increase in grain N-content. Grain N-content in maize was increased by both DCD (41%) and DMPP (38%), but N uptake and yield were improved (40 and 38%, respectively) by DCD only. DCD and DMPP remained equally effective in enhancing yield in grasslands (38 and 37%, respectively); however, DCD significantly increased plant N-uptake and grass biomass by 39 and 38%, respectively, while DMPP had shown a nonsignificant effect. The use of DMPP in vegetables increased biomass by 37%. DCD reduced soil NO_3_^−^-N content by 14% in rice fields but remained ineffective in influencing NO_3_^−^-N in soils under other crops included in this meta-analysis. DMPP lowered soil NO_3_^−^-N in fields of wheat (27%) and vegetables (30%). DCD reduced NH_4_^+^-N in rice fields by 31%, but its effect on soil NH_4_^+^-N content in all other crop fields was nonsignificant. DMPP decreased soil NH_4_^+^-N by 27 and 32% in vegetable fields and grasslands, respectively. Conversely, it enhanced NH_4_^+^-N content in soil by 56% under maize crop (Fig. 4).

### Effect of NIs and fertilizer type

**Fig. 5:**
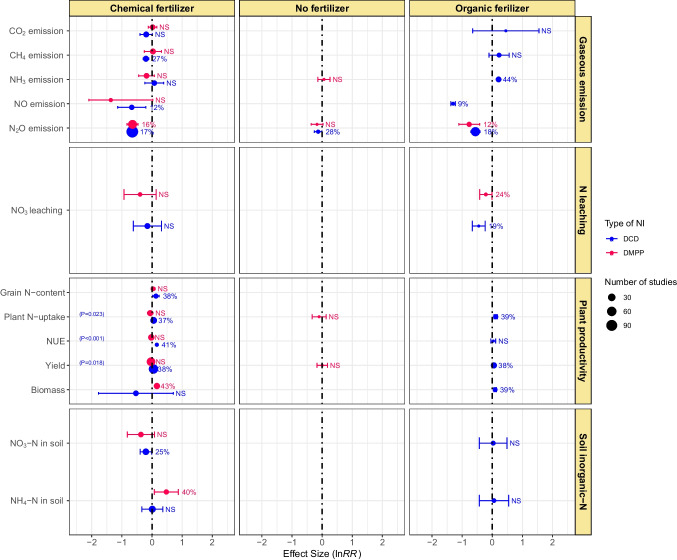
Effect of fertilizer type on DCD and DMPP efficacy in gaseous emissions, N leaching, plant productivity, and soil inorganic-N. Variables are considered significant if error bars do not overlap with zero. Error bars represent 95% CI. Variables are considered to be significant if error bars do not overlap with zero and are denoted in percent change (%) in effect size. Otherwise, NS shows a nonsignificant difference. Blue color represents the DCD, and red color represents DMPP treatments. The *p*-value inside each box denotes a significant difference between DCD and DMPP treatments, while nonsignificant differences show no *p*-values

The efficacy of the NIs significantly depended on the fertilizer type (organic, chemical, and no fertilizer) for reducing N losses, increasing plant productivity, and soil inorganic N status (Fig. 5). DCD application did not affect CO_2_ emission under all fertilizer types. However, it decreased CH_4_ emission by 27% in soils receiving chemical fertilizers, NO emission from both the chemical and organic fertilizers applied soils (9 and 12%, respectively), and N_2_O emission from unfertilized soil (28%), organic fertilizers (17%) and chemical fertilizers (18%) applied soils. However, DCD increased NH_3_ emission (44%) from soils receiving organic fertilizers. DMPP remained ineffective in reducing CO_2_, CH_4_, and NH_3_ emissions from chemical fertilizers applied to soils and NH_3_ and N_2_O from unfertilized soils. However, DMPP reduced N_2_O emissions by 12 and 16%, respectively, from the soils receiving organic and chemical fertilizers. Overall, DCD and DMPP had no significant effect on NO_3_^−^ leaching. However, individually NO_3_^−^ leaching was decreased by 19 and 24%, respectively, with DCD and DMPP application to soils receiving organic fertilizers. However, the NIs remained ineffective in reducing NO_3_^−^ leaching from soils receiving chemical fertilizers (Fig. 5).

The application of DCD along with organic fertilizers improved plant N-uptake by 39% and crop yield by 38% but remained ineffective in increasing NUE (Fig. 5). DMPP had nonsignificant effects on plant-N-uptake and crop yield in soils without any fertilizer. In chemical fertilizer-amended soils, DCD increased grain N-content, plant N-uptake, NUE, and yield by 38, 37, 41, and 38%, respectively; however, DMPP remained ineffective. The difference between the NIs was significant regarding their effect on plant N-uptake (*p* = 0.023), NUE (*p* < 0.001), and grain yield (*p* = 0.018). Soil inorganic-N (NO_3_^−^-N, NH_4_^+^-N) contents were not affected by DCD application in organic fertilizers amended soils. In soils receiving chemical fertilizers, DCD reduced soil NO_3_^−^-N content by 25% but showed a nonsignificant effect on soil NH_4_^+^-N content. On the other hand, DMPP had a nonsignificant effect on soil NO_3_^−^-N but escalated soil NH_4_^+^-N by 40% (Fig. 5).

### Effect of NIs and experiment type

**Fig. 6:**
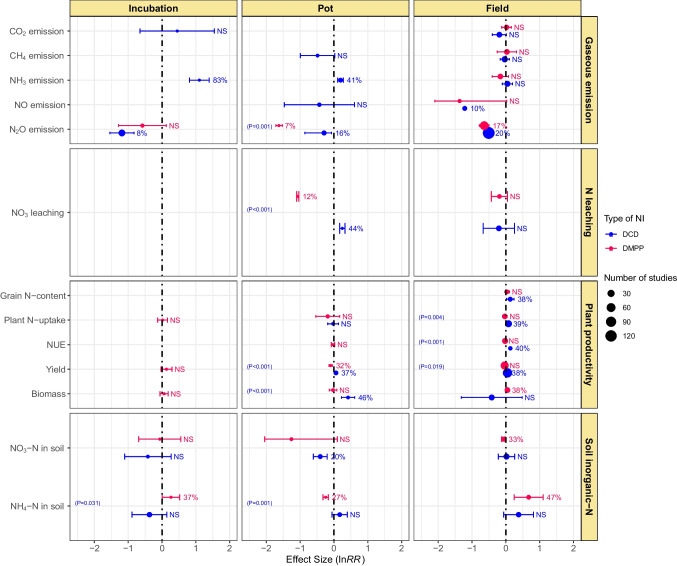
Effect of experiment type on DCD and DMPP efficacy in gaseous emissions, N leaching, plant productivity, and soil inorganic-N. Variables are considered significantly different if error bars did not overlap with zero. Error bars represent 95% CI. Variables are considered significantly different if error bars did not overlap with zero and are denoted in percent change (%) in effect size. Otherwise, NS shows a nonsignificant different. Blue color represents the DCD, and red color represents DMPP treatments. The *p*-value inside each box denotes a significant difference between DCD and DMPP treatments, while nonsignificant differences show no *p*-values

The effect of NIs as a function of experiment type (field, pot, and incubation) on gaseous emissions, N leaching, plant productivity, and soil inorganic-N status is presented in Fig. 6. Under field conditions, neither of NIs had a significant effect on CO_2_, CH_4_, and NH_3_ emissions from soils. However, under incubation and pot experiments, DCD increased NH_3_ emissions by 83 and 41%, respectively. Among the three experiment types, under field conditions, only the DCD application reduced NO emission (10%) and DCD caused a higher reduction in N_2_O emission than DMPP in all types of experiments. These differences were significant between both NIs under the pot as well as field conditions. Both NIs remained ineffective in reducing NO_3_^−^ leaching under field conditions. While in pot experiments, the NIs showed a contrasting effect on NO_3_^−^ leaching: DCD increased NO_3_^−^ leaching by 44%, whereas DMPP reduced NO_3_^−^ leaching by 12% (Fig. 6).

Only DCD improved grain N-content (38%), plant N-uptake (39%), NUE (40%), and yield (38%) under field experiments (Fig. 6). DMPP remained ineffective except for biomass which increased by 38%. In pot studies, DCD improved yield and biomass by 37 and 46%, respectively but showed a nonsignificant effect on plant N-uptake. On the other hand, DMPP increased grain yield by 32% but remained ineffective for plant N-uptake, NUE, and biomass in pot experiments. Similarly, the positive effect of DMPP was not found on plant N-uptake, yield, and biomass. The use of DCD reduced soil NO_3_^−^-N by 20% in pot experiments but remained ineffective in field and incubation experiments. Conversely, DMPP reduced soil NO_3_^−^-N by 33% in field experiments but had shown nonsignificant effects in incubation and pot experiments. DCD did not exhibit any significant effect on NH_4_^+^-N content in the soil, whereas DMPP increased soil NH_4_^+^-N in the following decreasing order: field experiment (47%) > incubation experiment (37%) > pot experiment (27%) (Fig. 6).

### Effect of NIs and soil texture

**Fig. 7:**
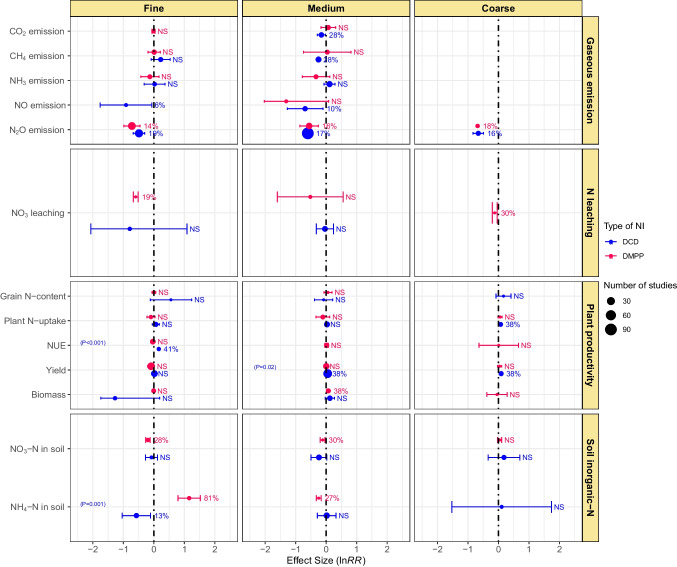
Effect of soil texture on DCD and DMPP efficacy in gaseous emissions, N leaching, plant productivity, and soil inorganic-N. Variables are significantly different if error bars did not overlap with zero. Error bars represent 95% CI. Variables are considered significantly different if error bars did not overlap with zero and are denoted in percent change (%) in effect size. Otherwise, NS shows a nonsignificant different. Blue color represents the DCD, and red color represents DMPP treatments. The *p*-value inside each box denotes a significant difference between DCD and DMPP treatments, while nonsignificant differences show no *p*-values

Gaseous N emissions from different textured agricultural soils, i.e., coarse, medium, and fine, differed greatly in response to the NIs application (Fig. 7). Both the NIs exhibited nonsignificant effects on CO_2_, CH_4_, and NH_3_ emissions in fine-textured soils, and DMPP did so in medium texture soils as well. In medium-textured soils, DCD mitigated the emission of both CO_2_ and CH_4_ by 28%, while its effect on NH_3_ was nonsignificant. In all the soil textures, DCD and DMPP were equally effective in reducing N_2_O emissions, ranging from 14 to 19%. The application of DCD in medium and fine-textured soils minimized NO emissions by 10 and 6%, respectively. This study finds a nonsignificant effect of DCD on NO_3_^−^ leaching in fine and medium-textured soils. However, DMPP reduced NO_3_^−^ leaching by 30 and 19% in coarse and fine-textured soils but proved ineffective in medium-textured soils (Fig. 7).

Wide variations were observed regarding plant productivity indices in response to NIs application in different soil textures. Both plant N-uptake and yield were improved by 38% with the use of DCD in coarse-textured soils; however, DMPP remained ineffective. In fine-textured soils, DCD significantly increased NUE by 41%, while all other plant productivity indicators included in the meta-analysis were not influenced significantly by either of the NIs. In medium-textured soils, DCD improved grain yield by 38%, whereas DMPP improved biomass yield by 38%, however, all other effects remained nonsignificant (Fig. 7). Application of DCD exhibited nonsignificant effects on soil NO_3_^−^-N content in all soil textures. On the other hand, DMPP reduced NO_3_^−^-N in soil by 28 and 30% in fine and medium-textured soils, respectively, but had a nonsignificant effect in coarse-textured soils. DCD decreased soil NH_4_^+^-N by 13% in fine-textured soils and showed nonsignificant effects in coarse and medium-textured soils. On the other hand, DMPP increased soil NH_4_^+^-N by 81% in fine-textured soils. Nonetheless, it reduced soil NH_4_^+^-N by 27% in medium-textured soils (Fig. 7).

### Effect of NIs and soil pH

**Fig. 8:**
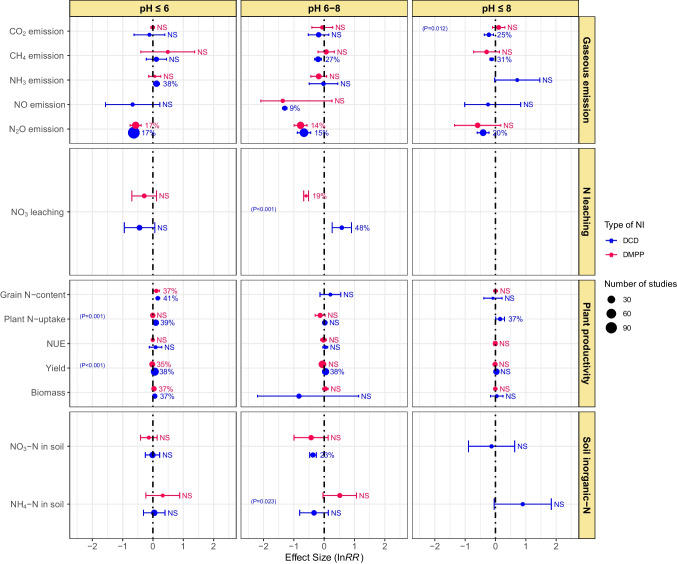
Effect of soil pH on DCD and DMPP efficacy in gaseous emissions, N leaching, plant productivity, and soil inorganic-N. Variables are significantly different if error bars did not overlap with zero. Error bars represent 95% CI. Variables are considered significantly different if error bars did not overlap with zero and are denoted in percent change (%) in effect size. Otherwise, NS shows a nonsignificant difference. Blue color represents the DCD, and red color represents DMPP treatments. The *p*-value inside each box denotes a significant difference between DCD and DMPP treatments, while nonsignificant differences show no *p*-values

Results pertinent to the effect of NIs on gaseous emissions and N leaching in croplands with different soil pH types, i.e. acidic (pH ≤ 6), neutral (pH 6–8), and alkaline (pH ≥ 8), are presented in Fig. 8. DCD did not affect CO_2_ and CH_4_ emissions from acidic soils, while decreasing CH_4_ emission by 27% in neutral soils and similarly decreasing CO_2_ and CH_4_ emissions by 26 and 31%, respectively, in alkaline soils. DMPP did not affect CO_2_ and CH_4_ emissions under different pH types. DCD affected NH_3_ emission only in acidic soil and increased it by 36%, whereas DMPP had shown a nonsignificant effect on NH_3_ emission in soils of different pH. DCD reduced NO emissions by 9% from neutral pH soils, while DMPP showed a nonsignificant impact. In acidic and neutral soils, DCD and DMPP were equally effective in mitigating N_2_O emissions, and emission reductions ranged from 14 to 17%. However, DCD reduced N_2_O emission in alkaline soils by 20%, whereas DMPP remained ineffective. Both NIs had a nonsignificant effect on NO_3_^−^ leaching from acidic soils. In neutral soils, however, the NIs showed converse effects on NO_3_^−^ leaching: DMPP reduced NO_3_^−^ leaching (by 19%), whereas DCD increased NO_3_^−^ leaching (by 48%) (Fig. 8).

In acidic soils, the use of DCD enhanced grain N-content, plant N-uptake, grain yield, and biomass by 41, 39, 38, and 37%, respectively (Fig. 8). Similarly, DMPP increased grain N-content by 41%, grain yield by 35%, and biomass yield by 37% in acidic soils. However, both NIs remained ineffective in improving NUE in acidic soils. In alkaline soils, except for plant N-uptake which increased by 37% with the application of DCD, all the productivity indicators were not affected by either of the NIs. Likewise, except for the 38% increase in grain yield by DCD in neutral pH soils, all plant productivity parameters were not influenced by either of the NIs. Both the NIs remained ineffective regarding the change in soil inorganic-N (NO_3_^−^-N and NH_4_^+^-N) in acidic soils. Concerning NH_4_^+^-N and NO_3_-N content in soils, only DCD resulted in higher NH_4_^+^-N content (23%) in neutral soils (Fig. 8).

## Discussion

### Effect of NIs on gaseous emissions

Mitigating gaseous emissions (i.e., CH_4_, CO_2_, NO, N_2_O, and NH_3_) from agricultural soils using NIs and their consequences on plant productivity have been extensively studied worldwide (Li et al. 2018; Qiao et al. 2015; Scheer et al. 2014; Sha et al. 2020; Wu et al. 2021; Xia et al. 2017; Yang et al. 2016b). There is still a debate on the efficacy of various NIs in reducing these gaseous emissions in relation to different soil and plant factors, particularly at the field level. We conducted this comprehensive meta-analysis to evaluate the effect of DCD and DMPP (the most commonly used NIs in agriculture) on gaseous emissions, N-leaching, plant productivity, and changes in soil inorganic-N status in relation to different crop and soil factors (crop type, fertilizer type, experiment type, soil texture type, and soil pH).

In general, NIs were ineffective in mitigating CO_2_, CH_4_, and NH_3_ emissions from croplands; however, they decreased NO and N_2_O emissions significantly. Among the NIs, in general, DCD mitigated N_2_O and NO emissions more effectively than DMPP (Figs. 3 and 4). Akiyama et al. (2010) conducted a meta-analysis and found that DCD was more effective than DMPP in mitigating N_2_O emissions. Yang et al. (2016b) also reported a significant reduction in N_2_O emissions amounting to 44 and 47% by DCD and DMPP, respectively. Similarly, Gao et al. (2021) reported an inhibition in N_2_O by 30 and 60% using DCD and DMPP, respectively. As nitrification and subsequent denitrification are the principal pathways for producing N_2_O and NO in agricultural systems, the NIs reduce N_2_O production by suppressing these processes (Kim et al. 2012).

Application of inhibitors mainly lowers NO_3_^−^-N availability for soil denitrification, thereby reducing N_2_O emissions (Benckiser et al. 2013). DCD effectively mitigated NO and N_2_O emissions from organic fertilizer-amended soils. In chemical fertilizer-amended soils, both NIs effectively reduced N_2_O emissions under field conditions (Figs. 5 and 6). Our findings further showed that texture type substantially influences the efficacy of NIs for N-emissions from soils. Both NIs effectively mitigated N_2_O emissions in coarse-textured soils. They also reduced N_2_O in fine-textured soils but had nonsignificant effects on CO_2_, CH_4_, and NH_3_ emissions. In medium-textured soils, DCD effectively reduced CO_2_, CH_4_, NO, and N_2_O emissions (Fig. 7). In coarse-textured soils, NIs increased the NH_4_^+^-N by delaying the process of denitrification (Cui et al. 2021). The reason behind could be the low physicochemical interaction in coarse texture soils due to low organic matter contents, while in heavy-textured soils, the higher organic matter and clay contents increased the activity of *Nitrosomonas* sp. which reduced the effectiveness of the NIs (Barth et al. 2019). However, further research is warranted to explore the mechanisms behind the varying effects of NIs on N emissions in different texture types.

DCD was found to increase NH_3_ emissions in vegetables, rice, and grasses but reduced its emission from wheat-cropped soils (Fig. 4). Similarly, in pot and incubation studies, DCD significantly increased NH_3_ emissions (Fig. 6). Kim et al. (2012) observed a significant increase in NH_3_ in urea-fertilized pastures and cropping soils by the application of NIs. Pan et al. (2016) found in a meta-analysis that DCD aggravates the release of NH_3_ by 22–220%, but DMPP had nonsignificant effects. Wu et al. (2021) also found that NIs increased NH_3_ volatilization by 35.7%, and volatilization varied greatly with NI type, soil pH, experimental method, and fertilizer type. The DCD and DMPP increased NH_3_ emissions by 27.4 and 43.2%, respectively (Gao et al. 2021). Similarly, Qiao et al. (2015) reported a 33–67% increase in NH_3_ emissions from agricultural systems by applying NIs. As the main principle, NIs interfere with the nitrification process, and NH_4_^+^ stays for a prolonged time in the soils. Thus, the longer the exposure of NH_4_^+^ to the soil environment, the higher chance of NH_3_ volatilization into the atmosphere (Soares et al. 2012). The solution to reduce NH_3_ volatilization from soil is demonstrated by many researchers including the use of slow release fertilizers, coated fertilizer, and biofertilizers. In a recent study, Xue et al. (2021) used the *Bacillus amyloliquefaciens* biofertilizer on alleviating ammonia volatilization in alkaline farmland soil. Biofertilizer treatment reduced ammonia volatilization by 68 percent, increasing crop production and nitrogen recovery by 19% and 19%, respectively, when compared to conventional fertilizer.

Converse to the finding of this study, the meta-analysis by Yang et al. (2016b) revealed an 8.7% reduction in CO_2_ emission with the use of DMPP. Likewise, Weiske et al. (2001) reported a significant reduction in mean CO_2_ emissions over three years by DMPP application to soil. This effect of DMPP is generally ascribed due to altered rates of C-mineralization in soil. Weiske (2001) observed a 28% reduction in CH_4_ emissions by DMPP, suggesting that it might stimulate CH_4_ oxidation. There exist many similarities between ammonium monooxygenase (AMO) and methane monooxygenase (MMO). Suppressing the activity of AMO by NIs might have improved the activity of MMO, thus facilitating the oxidation of CH_4_ (Wang et al. 2016). In contrast, Ménendez et al. (2012) and Huérfano et al. (2022) reported that DMPP is ineffective in reducing CO_2_ and CH_4_ emissions at different soil water and temperature regimes. However, the underlying mechanism needs further investigation. Gao et al. (2021) found a decrease in CH_4_ by 11% with DCD, and it is in line with the findings of this study.

Both NIs remained equally effective in mitigating N_2_O emissions from acidic soils. Likewise, DCD decreased CO_2_, CH_4_, and N_2_O emissions from alkaline soils, while DMPP remained ineffective. Both NIs reduced N_2_O emissions but had no significant impact on the release of CO_2_ and NH_3_ from neutral pH soils (Fig. 8). Kim et al. (2012) observed more NH_3_ emissions from soils with high pH irrespective of crop type and land use. Under alkaline conditions (pH ≥ 7.6), NH_4_^+^ ions dissociate into NH_3_ and thus favor NH_3_ volatilization (Francis et al. 2008). Unfortunately, there is still room for more study in this field because the relative efficacy of DCD and DMPP under various soil pH types has not been properly examined (Tufail et al. 2022).

### Effect of NIs on NO_3_^−^ leaching

NIs suppress nitrification and thus are expected to minimize subsequent denitrification and NO_3_^−^ leaching from soils (Norton and Ouyang, 2019; Subbarao et al. 2006). According to this meta-analysis, the overall effects of NIs were nonsignificant to NO_3_^−^ leaching from croplands. The effect of DCD and DMPP differed significantly among different crop types because of the large variance associated with the effect sizes. DCD reduced NO_3_^−^ leaching in grasses, while DMPP did so in maize crop. Conversely, NO_3_^−^ leaching was increased (39–49%) in wheat, maize, and other crops by DCD (Fig. 4). Both NIs significantly reduced NO_3_^−^ leaching from organic fertilizer-amended soils but remained ineffective in soils amended with chemical fertilizers (Fig. 5). However, Yang et al. (2016b) found in a meta-analysis that DMPP could effectively reduce soil NO_3_^−^-N leaching when applied with urea in neutral soils. This discrepancy between the meta-analysis studies might have occurred due to variations in the number of observations. For instance, the number of observations in our meta-analysis was greater (*k* = 220 for DMPP, 430 for DCD) as compared to an earlier study (*k* = 113 for DMPP, 185 for DCD) by Yang et al. (2016b).

In pot studies, contrasting effects of both NIs were observed, i.e., DMPP reduced, whereas DCD increased NO_3_^−^ leaching. Both the NIs did not exhibit any effect on NO_3_^−^ leaching under field conditions (Fig. 6). DCD had a nonsignificant effect on NO_3_^−^ leaching from fine and medium-textured soils. Conversely, DMPP reduced N leaching from fine and coarse-textured soils but had no effect in medium-textured soils (Fig. 7). Soil with coarse texture are more prone to leaching than fine texture soils, but more microbial biomass is present in fine-textured soils; therefore, DMPP reduced the N leaching from fine-textured soils. Both NIs had nonsignificant effects on N leaching from acidic soils. Nevertheless, DMPP reduced while DCD increased N leaching from neutral pH soils (Fig. 8). Our results are similar to Yang et al. (2016a, b), and they reported the higher effectiveness of DMPP over DCD in reducing soil NO_3_^−^-N leaching in neutral soils. However, future research is needed to do for understanding the whole mechanism.

### Effect of NIs on plant productivity

The management and environmental factors significantly influence the effectiveness of NIs. The findings of our meta-analysis demonstrated that using NIs in combination with chemical N-fertilizer could be an effective strategy to improve NUE and crop yields. This fact was further supported by the highly consistent effects of the NIs on a range of soil and crop management factors evaluated in the present study. In our meta-analysis, DCD increased grain-N and crop yield in rice and maize, and also increased plant N-uptake and biomass in grasses. Neither of the NIs improved wheat productivity but remained equally effective to enhance grass yield. On the other hand, DMPP improved rice yield, maize grain-N, and biomass yield in vegetables but did not significantly influence N-uptake and biomass in grasses (Fig. 4). Inhibiting the process of nitrification until the log phase of crop growth provides a better opportunity to absorb NO_3_^−^ thereby increasing NUE (Akiyama et al. 2010; Saud et al., 2022). We found higher NUE with the use of NIs, which agreed with the findings of Abalos et al. (2014), Qiao et al. (2015), Xia et al. (2017), and Li et al. (2018). Abalos et al. (2014) also found that the application of NIs along with fertilizer resulted in improved productivity of forages and cereal crops than fertilizer alone. They further stated that NIs increased NUE in forages, cereal, and vegetables/industrial crops. However, the effect was significantly higher for forages than cereals regarding productivity and NUE. One possible reason might be the higher N application in cereals compared to vegetables and forages, as DCD is relatively more effective under medium to high N application rates. Moreover, cereals are mainly harvested for grains, but aboveground biomass is more responsive to NIs than grain yield (Yang et al. 2016b).

Significant differences also existed among NIs regarding their effect on plant productivity in different fertilizer types. DCD improved plant N-uptake and crop yield but did not influence NUE in organic fertilizer-amended soils. On the other hand, DCD significantly increased grain-N, plant N-uptake, NUE, and grain yield in soils with chemical fertilizer, while DMPP showed no effect (Fig. 5). Yang et al. (2016b) found DCD to be more effective in improving crop yields when applied with organic or chemical N sources as compared to DMPP which had nonsignificant effects. DCD increased plant productivity and NUE, but DMPP remained ineffective except for biomass under field conditions. Both NIs improved grain yield but remained ineffective in influencing plant N-uptake in pot studies (Fig. 6). Application of NIs may aggravate N losses via NH_3_ volatilization under field and laboratory conditions, as indicated by a range of field and laboratory investigations (Kim et al. 2012). On the other hand, DCD improved plant N-uptake and grain yield in coarse-textured soil, while DMPP showed no significant effect. In fine-textured soils, DCD significantly increased NUE, but other plant productivity indicators were not influenced by both NIs. In medium-textured soils, DCD and DMPP improved grain and biomass yield, respectively (Fig. 7). In a meta-analysis, Abalos et al. (2014) found an average increase of 7.5% in crop yields and 12.9% in NUE as a result of using DCD and DMPP with a higher response on course-textured soils, as NIs prolong the detention of N in the soil as NH_4_^+^ thus providing more time for plants to uptake NH_4_^+^ (Kim et al. 2012). Abalos et al. (2014) observed a significantly lower response of NIs toward crop yield on fine-textured soils than medium or coarse-textured soils, although the effect was insignificant.

The efficacy of DCD and DMPP differed with the changes in soil pH: both NIs significantly improved plant productivity but remained ineffective to enhance NUE in acidic soils. In contrast, in alkaline soils, except for an increase in plant N-uptake by DCD, all productivity indices evaluated in the meta-analysis were not improved by both the NIs. In neutral pH soils, only DCD increased grain yield while other traits were not affected by both NIs (Fig. 8). Most probably, soil pH regulates the efficiency of NIs by affecting NH_3_ volatilization. The improved plant productivity on acidic and neutral soils by NIs might be attributed to their nonsignificant effects on N losses as NH_3_ emissions. However, Yang et al. (2016b) observed in their meta-analysis that DMPP only improved crop yields by 9.4% in alkaline soil, whereas DCD was equally effective in acidic and alkaline soils. Abalos et al. (2014) reported a positive response of NIs on crop productivity and NUE in three soil pH groups (i.e., ≤ 6.0, 6–8, and ≥ 8.0). However, the effect was the most significant when NIs were applied to acidic soils (pH ≤ 6.0). Linquist et al. (2013) found a higher positive response of NIs on N uptake and paddy yield in rice crop with high pH soils. In contrast, Abalos et al. (2014) observed a low response of NIs on NUE and crop yields on neutral and alkaline soils because of more N losses via NH_3_ volatilization.

### Effects of NIs on soil inorganic-N

Our meta-analysis showed that, in general, NIs effectively inhibit the process of nitrification as revealed by lower soil NO_3_^−^-N with subsequent increase in soil NH_4_^+^-N content (Fig. 3). DCD effectively reduced NO_3_^−^-N and NH_4_^+^-N content in rice crop only. DMPP significantly lowered soil NO_3_^−^-N in wheat and vegetables, whereas soil NH_4_^+^-N in grasses and vegetables. Conversely, DMPP enhanced soil NH_4_^+^-N content in maize crop (Fig. 4). For soil NO_3_^−^-N, DCD was found to be comparatively good NIs for chemical fertilizers applied soils (Fig. 5). Numerous investigations have documented that the efficiency of DCD and DMPP differed when applied with both organic and chemical fertilizers (Dai et al. 2013; Lei et al., 2022; Yang et al. 2016a). This discrepancy could be attributed to differential hydrolyzing rates of N sources to NH_4_^+^ form, available for nitrification in soil. Yang et al. (2016b) revealed that both DCD and DMPP, when combined with organic fertilizer or urea, proved equally effective in increasing soil NH_4_^+^-N content. NIs significantly limit the conversion rate of NH_4_^+^ to NO_3_^−^, increase the NH_4_^+^ in the soil profile, and ultimately increase soil NH_4_^+^-N contents. In case of organic fertilizers the NIs significantly reduced the population of AOB and amoA gene abundance (Tao et al. 2021). The organic fertilizer provides suitable alkaline conditions for NIs to reduce the population of AOBs (Lei et al., 2022).

NIs primarily hampers the microbial conversion of NH_4_^+^-N to NO_3_^−^-N, thus minimizing N losses through leaching (Benckiser et al. 2013; Meng et al. 2021). We found that DCD effectively reduced soil NO_3_^−^-N in post studies, while DMPP did so under field conditions (Fig. 6). DMPP reduced NO_3_^−^-N in fine and medium-textured soils. In contrast, DMPP significantly increased soil NH_4_^+^-N in fine-textured soils but reduced NH_4_-N in medium-textured soils (Fig. 7). Both NIs remained ineffective regarding the change in soil inorganic-N in acidic soils. DCD slightly reduced NO_3_^−^-N in alkaline soils while significantly reducing soil NO_3_^−^-N and NH_4_^+^-N content in neutral pH soils (Fig. 8). Gao et al. (2021) found that DCD and DMPP increased NH_4_^+^-N by 90.7 and 81.6% under incubation conditions whereas 46 and 44% under field conditions, respectively. They further reported decrease in NO_3_^−^-N by 45.5 and 70% under the laboratory and by 25.2 and 20.9% in the field with DCD and DMPP, respectively. Higher microbial activities in the field than laboratory conditions usually accelerate biodegradation rates of NIs, thereby declining their efficiencies (Kelliher et al. 2014).

## Conclusion

The meta-analysis undertaken here has extended our knowledge and concluded that the overall effect of NIs was insignificant in reducing CO_2_, CH_4_, and NH_3_ emissions. It further concludes that (i) DCD and DMPP remained equally effective in reducing N_2_O emissions; however, DCD reduced NO emission by 16%, whereas the effect of DMPP was nonsignificant. (ii) Although the effect of NIs was highly crop-specific, but the DMPP and DCD were highly effective in mitigating N_2_O emissions. (iii) DCD decreased CH_4_ emission by 27% in soils receiving chemical fertilizers, while DMPP remained ineffective in reducing CO_2_, CH_4_, and NH_3_ emissions from chemical fertilizers. (iv) The effect of soil texture and soil pH on NIs suggested that DCD and DMPP were equally effective in reducing N_2_O emissions in all the soil textures and from acidic and neutral soil. Taken together, these observations indicate our understanding of the role of DMPP and DCD in various agroecological scenarios. However, more research is needed to understand the role of NIs other than DCD and DMPP.

## Supplementary Information

Below is the link to the electronic supplementary material.

Supplementary file 1 (XLSX 63 kb)

Supplementary file 2 (PNG 78 kb)

High resolution image (TIFF 9228 kb)

## Data Availability

Not applicable.
