# Supplementary figures and images for: Mediation of gaseous emissions and improving plant productivity by DCD and DMPP nitrification inhibitors: Meta-analysis of last three decades
Source: Environ Sci Pollut Res Int. 2023 Mar 16;30(23):64719–35. doi: 10.1007/s11356-023-26318-5 (PMC10172236; doi:10.1007/s11356-023-26318-5)

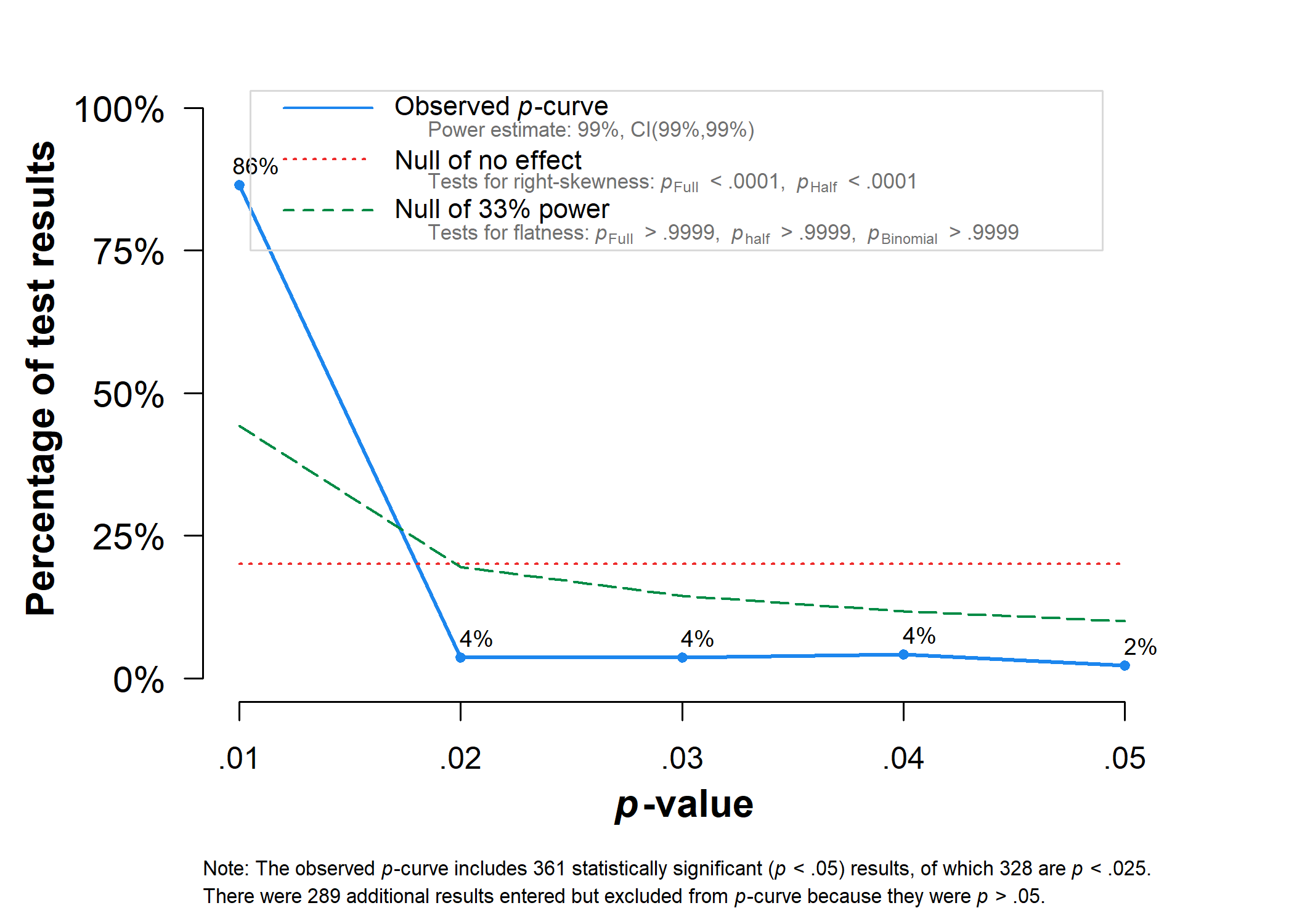

Supplement: Supplementary file 2 — (PNG 78 kb) [file 11356_2023_26318_Fig9_ESM.png]

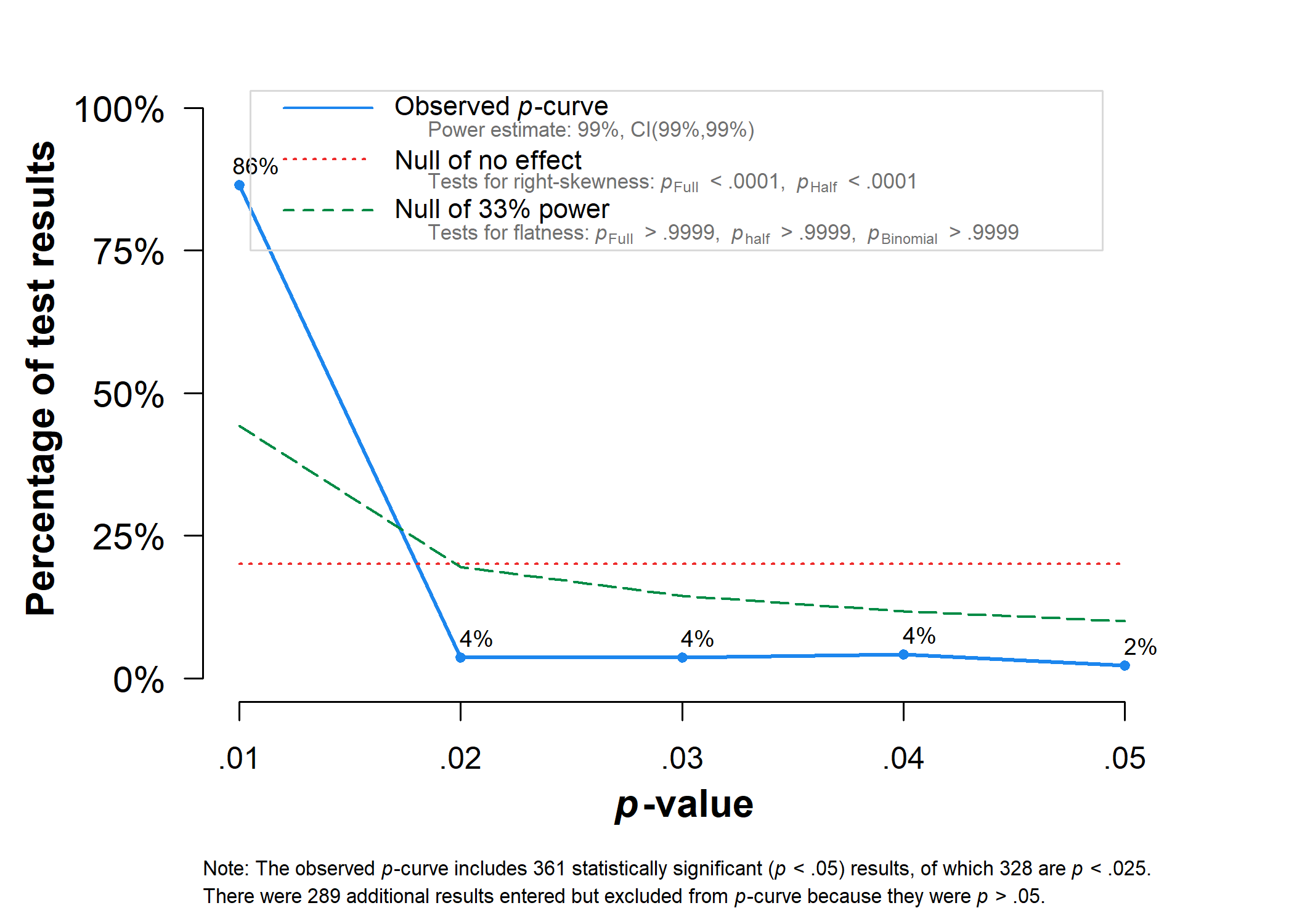

Supplement: Supplementary file 3 — High resolution image (TIFF 9228 kb) [file 11356_2023_26318_MOESM2_ESM.tiff]
